# Oxidative stability and elemental analysis of sunflower (*Helianthus annuus*) edible oil produced in Brazil using a domestic extraction machine

**DOI:** 10.3389/fnut.2022.977813

**Published:** 2022-09-30

**Authors:** David Johane Machate, Elaine S. P. Melo, Lincoln Carlos Silva de Oliveira, Danielle Bogo, Flávio S. Michels, Arnildo Pott, Leandro F. Cavalheiro, Rita de Cássia Avellaneda Guimarães, Karine de Cássia Freitas, Priscila Aiko Hiane, Anderson R. L. Caires, Marcelo Luiz Brandão Vilela, Rodrigo Juliano Oliveira, Valter Aragão do Nascimento

**Affiliations:** ^1^Graduate Program in Materials Science, Federal University of Mato Grosso do Sul, Campo Grande, Brazil; ^2^Group of Spectroscopy and Bioinformatics Applied Biodiversity and Health (GEBABS), Graduate Program in Health and Development in the Central-West Region of Brazil, Federal University of Mato Grosso do Sul, Campo Grande, Brazil; ^3^Chemistry Institute, Federal University of Mato Grosso do Sul, Campo Grande, MS, Brazil; ^4^Post-graduate Program in Health and Development in the Mid-West Region, Federal University of Mato Grosso do Sul, Campo Grande, MS, Brazil; ^5^Optics and Photonics Group, Institute of Physics, Federal University of Mato Grosso do Sul, Campo Grande, MS, Brazil; ^6^Graduate Program in Biotechnology and Biodiversity in the Central-West Region, Federal University of Mato Grosso do Sul, Campo Grande, MS, Brazil

**Keywords:** cold-press, vegetable oil quality, trace elements, oil quantity consumption, non-carcinogenic indices

## Abstract

The consumption of regular vegetable oils has been linked to energy acquisition, nutritional benefits, health improvement, and the regulation of metabolic diseases. This study evaluated fatty acids composition, physicochemical, thermal, oxidative, and optical properties, and quantified trace elements in the sunflower oil extracted by a domestic cold-press machine. The oil presented linoleic (54.00%) and oleic (37.29%) primary unsaturated fatty acids (91.67%), in which atherogenic (0.05), thrombogenic (0.16), hypocholesterolemic/hypercholesterolemic (21.97), peroxide (16.16), saponification (141.80), and relative density indices (0.92) demonstrated to be suitable for human consumption and possible health promotion. In addition, the concentrations of trace elements by ICP OES were ordered Zn > Fe > Al > Cu > Mn > Cr. Concentrations of Zn, Fe, Al, Cu, and Mn were lower than FAO/WHO and DRI/AI limits, while Cr concentrations exceeded the FAO/WHO limits, which can be used as an indicator of the polluted ambiance. Sunflower oil quantities daily consumption were calculated by taking into account non-carcinogenic risk (CR < 10^−4^), and total non-carcinogenic hazard index (HI < 1). Based on trace elements determined in this study, the suitable quantity of sunflower oil consumption varies according to individuals aged 8, 18, and 30 years and will be deemed 0.61, 1.46, and 1.65 g/kg, respectively, attending HI = 0.99 and CR < 10^−4^.

## Introduction

One of the three main energy sources for human life activities is edible vegetable oil, whose stages (field production, harvesting, processing, storage) must be monitored (1). In addition, vegetable oils are strictly associated with health promotion and metabolic diseases prevalence such as obesity, diabetes mellitus, cardiovascular (CVD), coronary heart diseases (CHDs), systemic inflammation, atherosclerosis, cancers, etc. (2). Benefits of vegetable oils are associated with fatty acids composing them: saturated (C6:0–C20:0, no double bonds) monounsaturated (C16:1–C20:1, one double bond) and polyunsaturated (C18:2–C24:6), two up to six double bonds) (2, 3), essential components: macro- and microelements, vitamins A, C, K, and E (tocopherols, tocotrienols), phytosterols, phytostanols, carotenoids, chlorophylls, polyphenols, flavonoids, acting as antioxidants and health promoting (4–6).

Most commercially available plant seed and fruit oils are extracted using petroleum solvents, with high toxicity levels for human health (7). Moreover, their essential components are removed during refining (chemical and physical) and bleaching processes, formatting short-chain compounds [esters, polymeric triacylglycerols, trans and free fatty acids, (hydro) peroxides, dienes and trienes, and others], which act as pro-oxidants (8). These substances reduce vegetable oil shelf life and vital properties, which decrease healthy and nutraceutical properties, and are associated with the occurrence of several metabolic diseases in consumers (9) due to the higher amount of free radicals increasing oxygen species (10).

Globally, the sunflower is the second most cultivated plant after maize, and its seeds are used to treat inflammatory, hypertensive, CVD, and other diseases (11). Additionally, its oil controls tumors, diabetes, cholesterol, cancers, hypertension, hypercholesterolemia, and CHD. These benefits are associated with the synergic action of tocopherols, phytosterols, terpenoids, polyphenols, carotenoids, alkaloids, amino acids, proteins, vitamins, oleic acid, antioxidants, and other anti-inflammatory substances (4, 12, 13). Due to reducing of the production of the reactive oxygen species (ROS) and reactive nitrogen species (RNS), consequently lowering disorder metabolites [superoxide ions (*${\text{O}}_{2}^{-}$), hydroxyl (OH^−^), hydrogen peroxide (H_2_O_2_), nitrate oxide (NO), etc.], which are linked with pro-inflammatory several metabolites [interleukin-1,−1β,−6,−8,−12 (IL-1,−1β,−6,−8,−12), Toll-like receptor 4 (TLR4), lipopolysaccharides (LPS, microbial component), hepatic nuclear factor-kappa B (NF-kB), tumor necrosis factor-alpha (TNF-α), inducible nitric oxide synthase (INOS), cyclooxygenase-2 (COX-2), lipoxygenase (LOX), cytochrome P450, nitric oxide, G protein-coupled receptor 120, etc.] potentially associated with oxidative stress and metabolic diseases due to high damage of cellular protein, lipids, and DNA (9, 14, 15).

Nevertheless, it is remarkable that food frying (pan and deep) using sunflower oil rich in linoleic fatty acid reduces its oxidation and thermal stability, in which various hazardous substances to human health form (16). To reverse this scenario, blending cold-pressed oils rich in natural antioxidants or their natural antioxidants [thymoquinone and tocopherols from black cumin (*Nigella sativa*)] with sunflower oil is highly recommended for oils stability and for consumers' health improvement (16–19).

In addition, vegetable oils are a source of macroelements such as sodium (Na), potassium (K), calcium (Ca), magnesium (Mg), phosphorus (P), and microelements such as iron (Fe), selenium (Se), manganese (Mn), chromium (Cr), zinc (Zn), aluminum (Al), barium (Ba), strontium (Sr), tin (Sn), copper (Cu), cobalt (Co), and thallium (Ti), which are essential or toxic for human health when consumed in large quantities (20, 21). In addition, some chemical elements, such as arsenic (As), cadmium (Cd) nickel (Ni), lead (Pb), mercury (Hg), and Cu are found in edible vegetable oils, which are toxic (22–24) and carcinogenic for consumers even at a low amount (25). Moreover, the presence of Ca, Co, Mg, Fe, Zn, Cu, Mn, Sn, and Ni accelerate vegetable oils' oxidation affecting their flavor, freshness, storability, and toxicity (22, 26). Therefore, cold pressing and filtration are the alternatives of the several methods to obtain healthy edible vegetable oils associated with good fatty acids and antioxidants composition, and lower amounts of hazardous chemical elements (27–29).

Interestingly, it is increasing the consumption of the unrefined edible vegetable oils, including sunflower oils obtained by cold pressed due to the high amount of natural antioxidants (tocopherols, phytosterols, carotenoids, etc.), waxes, while presenting low content of free fatty acids and phospholipids (30, 31). In addition, some studies have reported nutritional qualities correlated with physicochemical properties, trace elements, fatty acid composition, and essential components in sunflower oil obtained by cold pressing (32). Therefore, a domestic cold-press machine is mostly recommended to obtain healthy edible vegetable oils, due to their higher amount presence of antioxidants and fatty acids, moreover, with a lower amount of heavy metals contaminants (20).

Although some studies have been carried out using different types of oils and extraction conditions, there is scarce information in the literature on the quality and mineral composition of sunflower oil extracted by a domestic cold-press machine, as well as risk assessment for human health due to the ingestion of this type of oil containing metals.

Motivated by the manuscript published by Melo et al. (20), which demonstrated that cold-extracted oils maintain their quality and chemical composition, this study aimed to evaluate for the first time the fatty acids composition, physicochemical and optical properties, thermal and oxidative stability of sunflower oil samples cold-press extracted using a domestic machine. In addition, the chemical elements Mg, Cr, Mn, Fe, Co, Ni, Cu, Zn, Cd, Al, Pb, As, and Se in sunflower oils were quantified using inductively coupled plasma optical emission spectroscopy (ICP OES) and the results were compared with DRI/AI^*^ and FAO/WHO parameters. The fatty acid composition, physicochemical and optical properties, and thermal and oxidative qualities evaluated in this oil were compared with previous results in the literature.

## Materials and methods

### Sunflower seed, oil preparation, seed and oil moisture, and lipid quantification

Sunflower seeds were obtained from 10 farms in Campo Grande, Mato Grosso do Sul state, Brazil, in September 2020. Seeds were mixed and immediately dried in an air circulation oven at 40°C for 48 h. We collected crude oil from dried hull seeds using a domestic cold-press machine extractor equipped with a stainless continuous screw and drainage hole with an internal filter (Yoda Nut & Seed Cold Press Oil), Extractor-Gourmet Extractor, oil Natural, Homeup, (Yoda Europe, Cluj-Napoca, Romania). Immediately, the oil was placed into an amber and hermetic glass bottle and then used for analysis.

Moisture content was measured using milled seeds (1.0 g) subjecting in the over at 105°C for 60 min, then the sample was rested in a desiccator, until achieving constant weight. For the filtered sunflower oil (1.4 g), relative humidity was determined by the Karl Fischer technique (KEM MKC-610 Karl Fischer Moisture Titrator, Japan).

Free fatty acids (FFAs) were extracted using a Soxhlet extractor with petroleum ether as solvent at 60°C for 6 h (33).

### Methylation and fatty acid profile

Fatty acid methyl esters (FAMEs) of the sunflower oil were obtained at ambient temperature. The samples (157 mg) were weighed into assay tubes, saponified with methanolic NaOH 0.5 N (4 ml), esterified with a mixture of H_2_SO_4_ and NH_4_Cl in methanol (5 ml), then saturated with NaCl (4 ml), and finally dissolved in hexane (5 ml) (34).

FAMEs were analyzed using a gas chromatograph (model CP-3800, Varian, Santa Clara, CA, USA) equipped with a flame ionization detector, a split/splitless injector, and a stationary phase fused silica capillary column of polyethylene glycol (CARBOWAX 20 M, length 30 m × 0.25 mm, Quadrex, Santa Clara, CA, USA). Operational parameters were followed for chromatography: the injector and detector temperatures were 250°C. The column temperature was programmed to 80°C for 2 min, followed by a ramp of 4°C /min up to 220°C and kept for 13 min; hydrogen carrier gas with 1 mL min^−1^ flow and injection volume 1 μL. Retention times were compared with the respective methyl ester standards (Supelco, F.A.M.E. mix C4:0 to C24:0, Sigma-Aldrich, Darmstadt, DA, Germany) (35).

### Fatty acids nutritional quality indices

The nutritional quality of sunflower oil was evaluated according to its fatty acids composition assessed by three following indices

Atherogenicity index (AI) (36).


(1) $$\text{AI }=\;\frac{\text{C}12:\text{0 }+\left(\text{4}\times \text{C}14:0\right)\;+\text{ C}16:\text{0}}{\sum \text{MUFA }+\;\sum \text{PUFA}}$$

Thrombogenicity index (TI) (36)


(2) $$\text{TI = }\frac{\text{C}14:0\;+\text{ C}16:\text{0 }+\text{ C}18:0}{(0.5\times \sum \text{MUFA})\;+(0.5\;\times \sum \omega 6\text{) }+\;(3\;\times \;\sum \omega 3\text{)}}$$

Fatty acids hypocholesterolemic/hypercholesterolemic (HH) ratio (37)


(3) $$\text{HH = }\frac{\text{C18}:1\omega 9\;+\text{ C}18:2\omega 6\;+\text{ C}20:4\omega 6\;+\text{ C}18:3\omega 3\;+\text{ C}20:5\omega 3\;+\text{ C}22:5\omega 3+\text{C}22:6\omega 3}{\text{C}14:0\;+\text{ C}16:0}$$

### Identity and quality characteristics of sunflower oil

Sunflower oil characterization was conducted according to the American Oil Chemist's Society (38), in triplicate, for qualification parameters: acidity (Ca 5a-40) and peroxide indices (Cd 8-53); identity parameters: relative density (Cc 10a-25), iodine-Wijis method (d 1-25), and saponification values (Cd 3-25).

### Determination of oxidative stability

Sunflower oil oxidative stability was determined by the Rancimat method (873 Metrohm Co, Basel, Switzerland) by accelerated oxidation according to the European Union standardized standard EN 14112 (39). The analyses were conducted subjecting 3.0 g of oil at a constant temperature of 110°C under an airflow rate of 10 L h^−1^ through the samples, and then into a measuring vessel containing 50 ml ultrapure water Mill-Q in which the conductivity generated by volatile products during the oil decomposition was measured as a function of time.

### Thermal analyses: Thermogravimetric analysis/derivative thermogravimetry, and differential scanning calorimetry

Thermogravimetric analysis (TGA)/derivative thermogravimetry (DTG) curves were obtained using TGA-Q50 (TA Instruments, New Castle, DE, USA). Samples of sunflower oil (6.0 mg) were added into a platinum pan from 10 to 550°C at a heating rate of 2°C min^−1^ under inert nitrogen and synthetic air atmosphere gases at a flow rate of 60 ml min^−1^. In addition, DSC curves were conducted with DSC-Q20 equipment with RCS90 coupled with a cooling system (TA Instruments). The DSC curves were obtained in a calorimeter model DSC-Q20 coupled with an RCS90 refrigeration system (TA Instruments). Approximately 3.0 mg of sunflower oil was used for analysis using aluminum crucibles (Tzero standard) as support and reference, at a heating rate of 10°C min^−1^, heating cycle followed by cooling at temperatures between −80°C and 25°C, under an inert nitrogen atmosphere with a flow rate of 60 ml min^−1^.

Curves were obtained from TGA/DTG and DSC data, which were generated by Universal Analyzes 2000 software version 3.7A (TA Instruments).

### Optical molecular analyses: UV-Visible absorption and fluorescence spectroscopy

Sunflower oil was diluted in HPLC grade hexane (spectroscopic grade 99.9%) at a concentration of 1 × 10^−3^ g L^−1^. UV-Visible absorption measurements were made using a Lambda 265 UV/Vis spectrophotometer (Perkin Elmer, Waltham, MA, USA), and the spectra were collected in the 200 to 800 nm range.

Excitation-emission matrix fluorescence maps were obtained using a spectrofluorometer (FluoroMate FS-2, SCINCO). The excitation-emission maps were obtained by exciting the samples between 240 and 450 nm in steps of 5 nm and collecting the emission from 250 to 750 nm in 1 nm steps.

For the UV/Vis absorption and fluorescence measurements, the diluted sunflower oils were placed into a four-polished-sided quartz cuvette with a 10 mm optical path.

### Extraction induced by emulsion breaking procedure and trace elements quantification

The extraction induced by emulsion breaking was performed according to Carneiro et al. (40) with adaptations. A falcon tube containing 3.0 ml of crude sunflower oil and 3.0 ml of ethanol was shaken for 20 s in a vertex shaker, then 3.0 ml of ultrapure water (conductivity 18.2 MΩcm, Millipore, Biocel, Germany), 0.76 ml of Triton x-100, and 3.0 ml of HNO_3_ were added, and then mixed with a vortex mixer for 20 min.

The concentration of Al, Cr, Cu, Fe, Mn, and Zn in sunflower oil samples was obtained using an ICP OES with an Axial Plasma (iCAP 6000 Series, Thermo Scientific, Cambridge, UK). Standard solutions were prepared by diluting a standard multi-element stock solution (SpecSol, Quinlab, Jacarei, SP, Brazil) containing 1,000 mg L^−1^ of each element. Nine concentrations were used to build calibration curves for the quantitative analyses of oil. The concentration for the elements was 0.01–5.0 mg L^−1^ range. The setup of ICP OES instrumental conditions for elemental analyses was the same used by Melo et al. (20). Table 1 summarizes the operational condition used in the ICP OES apparatus analysis as wavelength, limit of detection (LOD), limit of quantification (LOQ), and correlation coefficient (R^2^) in the current study.

**Table 1:** The ICP OES operating conditions for analysis.

**Elements**	**Wavelength (nm)**	**LOD (mg/kg)**	**LOQ (mg/Kg)**	**Correlation (R^2^)**
Al	396.152	0.0114	0.0380	0.9995
As	193.696	0.0048	0.0159	0.9996
Cd	228.802	0.0007	0.0024	0.9994
Co	238.892	0.0016	0.0054	0.9995
Cr	425.435	0.0015	0.0049	0.9994
Cu	327.396	0.0014	0.0048	0.9995
Fe	259.940	0.0193	0.0644	0.9991
Mg	285.213	0.0675	0.2251	0.9984
Mn	257.610	0.0004	0.0012	0.9996
Ni	221.647	0.0023	0.0077	0.9994
Pb	220.353	0.0070	0.0233	0.9996
Se	196.026	0.0080	0.0267	0.9997
Zn	213.856	0.0018	0.0060	0.9991

One blank and nine calibration curves were generated using the following concentrations: 0.005, 0.01, 0.025, 0.05, 0.1, 0.25, 0.5, 1.0, and 2.0 mg kg^−1^ of each element standard.

An addition/recovery test for the elements under study was carried out in a sunflower oil sample by spiking 0.5 mg L^−1^ of each analyte. The method had a recovery interval of 80–110% for the spike of 0.5 mg L^−1^, to the established limit proposed by the Association of Official Analytical Chemists (41).

### Human health risk assessment

The concentration of the chemical elements in sunflower oil was compared with the FAO/WHO recommended intake standards and hazards quotient. The non-carcinogenic was calculated according to the equation adopted by Machate et al. (42). Cancer risk is the probability of an individual developing any cancer type over a lifetime due to a specific exposure to a hazardous mineral. The chronic daily intake dose (CDI) of carcinogenic elements (mg/kg/day) and slope factor (SF) of Cr is 0.5 mg/kg/day, according to Equation (4).


(4) Cancer Risk = CDI ×SF

Cancer risk is a sum of individual chemical elements in different exposure pathways to develop cancer in a person, which is the total cancer risk (CR). According to US UPA (43), acceptable values of cancer risk range from 10^−6^ to 10^−4^, while values > 10^−4^ are considered inadmissible.

The human health risk of trace element consumption dose was calculated on the chronic daily intake dose (CDI, mg/kg/day) for a chemical contaminant element in the sunflower oil intake quantity CDI calculated according to Equation (5).


(5) $${\text{CDI}}_{\text{oil}}\text{=}\frac{\text{C}\times \text{IR }\times \text{EF }\times \text{ED}}{\text{BW }\times \text{AT}}$$

where CDI_oil_ – chronic daily oil intake dose; C – chemical element concentration in the crude sunflower oil sample (mg kg^−1^); IR – ingestion rate g/day; EF – exposure frequency (365 days/year); ED – exposure duration; BW – body weight (kg), estimated 26, 62, and 70 kg for 8, 18, and 30 years old, respectively. AT – average time (ED × 365 days/year).

The risk to human health by the intake of trace element contaminated food was estimated using a hazard quotient (HQ), which is a ratio of CDI and chronic oral reference dose (RfD), determined by the following Equation (6):


(6) $$\text{HQ = }\frac{\text{CDI}}{\text{RfD}}$$

The RfD values were previously established by the Joint Food and Agriculture Organization/World Health Organization Expert Committee on Food Additive (44) and the United States Environmental Protection Agency (45). The RfD (mg/kg/day) values are: Al = 1.0; Cr = 0.003; Cu = 0.04; Fe = 0.7; Mn = 0.14; and Zn = 0.3 (46). As shown in Equation (6), hazard quotient toxic risk on each trace element and their sum, Equation (7) (total non-carcinogenic hazard index) HI < 1, safe food consumption, while HI > 1, health risk food consumption.


(7) $$\begin{array}{l} \text{HI}=\text{H}{\text{Q}}_{\text{Al}}{+\text{HQ}}_{\text{Cr}}{+\text{HQ}}_{\text{Cu}}{+\text{HQ}}_{\text{Fe}}{+\text{HQ}}_{\text{Mn}}{+\text{HQ}}_{\text{Zn}} \end{array}$$

## Results and discussion

### Oil preparation, sunflower seed, oil moisture, and lipid quantity

Sunflower seed yielded 260 mg g^−1^ (26%) of oil, lower than industrially obtained using petroleum solvents (36–50%) (47). However, oil extracted by cold-press yielded a higher amount of beneficial nutritional components such as tocopherols, phytosterols, terpenoids, phenolic acids, carotenoids, chlorophylls, antioxidants, waxes (C36–C48), oleic and linoleic fatty acids, phospholipids, unsaponifiable matters, and others; in contrast, the hazardous components (trace metals) were extracted in lower amounts (29, 48, 49). The cold-pressing technique is widely recommended because it provides oils with healthy components, including their by-products (oil cake) (32), of which adequate consumption is correlated with regulating metabolic diseases (12).

The moisture determination in sunflower seeds yielded 72.76 mg g^−1^ (7.28%) of dehydrated water, whereas dry matter was 927.24 mg g^−1^ (92.72%), better for fatty acid and essential components integrity, which are relevant for nutrition functionality (50). Sunflower oil moisture was 998 μg, (0.07%) of evaporated water, demonstrating the importance of this oil in food system quality, due to less susceptibility to oxidative stress and lipid oxidation by the action of pro-oxidants substances (51). Refined flour of sunflower seed (1.0 g) yielded 493.82 mg g^−1^ (49.38%) of FFAs, demonstrating its relevance for nutritional value and a good source of vegetable oil (29).

### Fatty acid profile

Table 2 presents the composition and quantity of fatty acids yielded in this study, with profiles in decreased order: linoleic (54.00%) > oleic (37.29%) > palmitic (4.13%) > stearic (3.17%) > behenic (0.55%) > araquidic (0.20%) > lignoceric (0.17%) > gondoic (0.13%) > docosadienoic = tricosylic (0.08%) > eicosapentanoic (0.06) > α-linolenic (0.05%) > palmitoleic = margaric (0.04%) > meristic (0.03%). In this study, most quantified fatty acids, linoleic/oleic ratios were proportionally shown at 1.44:1, better than oils extracted by n-hexane as solvent 3.24:1 (32, 52).

**Table 2:** Fatty acid composition of sunflower oil extracted by cold pressing.

**Fatty acids**	**Mean ±Standard deviation (%)**
Myristic (C14:0)	0.03 ± 0.00
Palmitic (C16:0)	4.13 ± 0.05
Palmitoleic (C16:1)	0.04 ± 0.00
Margaric (C17:0)	0.04 ± 0.00
Heptadecenoic (C17:1)	0.02 ± 0.00
Stearic (C18:0)	3.13 ± 0.08
Oleic (C18:1n9c)	37.29 ± 0.24
Linoleic (C18:2n6c)	54.00 ± 0.39
α-Linolenic (C18:3n3c)	0.05 ± 0.00
Araquidic (C20:0)	0.20 ± 0.00
Gondoic (C20:1)	0.13 ± 0.00
Eicosapentanoic (C20:5n3c)	0.06 ± 0.00
Behenic (C22:0)	0.53 ± 0.01
Docosadienoic (C22:2)	0.08 ± 0.00
Tricosylic (C23:0)	0.08 ± 0.00
Lignoceric (C24:0)	0.17 ± 0.01
Σ SFAs	8.33
Σ MUFAs	37.48
Σ PUFAs	54.19
Σ UFAs	91.67
Total FAs	100
Atherogenic index	0.05
Thrombogenic index	0.16
Hypocholesterolemic/hypercholesterolemic	21.97

Σ SFAs, sum of saturated fatty acids; Σ MUFAs, sum of monounsaturated fatty acids; Σ PUFAs, sum of polyunsaturated fatty acids; FAs, fatty acids; ND, non-detectable; defined as < 0.05%.

According to our results, the sunflower oil extracted using a domestic cold press machine yielded a higher amount of oleic acid and the highest hypocholesterolemic (HH) ratio (Table 2) compared with one that applied n-hexane (oleic, 19.81%; linoleic, 64.35%; HH, 0.16) (32), massively used by industry to obtain edible vegetable oils.

Therefore, long-term diets of vegetable oils rich in linoleic acid and the lowest hypocholesterolemic index are associated with the prevalence and incidence of several metabolic diseases (diabetes mellitus, coronary and inflammatory diseases, cancer, and obesity) (4, 6, 53). Moreover, in the human body, linoleic acid is converted into arachidonic acid (n-6 PUFAs), which is the pro-inflammatory precursor of prostaglandin and leukotriene synthesis at the cyclooxygenase (COX-2), lipoxygenase (LOX), and cytokines (TNF-α, IL-1, IL-1β, IL-6, IL-8, IL-12, NF-kB, NO, LTs cytochrome P450, protein-coupled receptor 120), which compete with n-3 PUFAs enzymes during the biosynthesis pathway of long- (LC-PUFAs) and very-long-chain fatty acids (VLC-FAs) associated with anti-inflammatory effects (9, 14, 15).

### Fatty acid nutritional quality indices and characteristics of sunflower oil

Table 2 depicts nutritional quality indices: atherogenicity index (AI), thrombogenicity index (TI), and hypocholesterolemic/hypercholesterolemic (HH) ratio calculated as 0.05, 0.16, and 21.97, respectively. Sunflower oil obtained by the cold-press machine presents better values associated with regulating several metabolic diseases for consumers than another extracted by petroleum solvent (54).

Table 3 presents physicochemical profiles revealing that this sunflower oil is suitable for human consumption, and its averages can be associated with oxidative stability, authenticity, quality, and identity (57). The iodine index (131.53) was found between the Codex Alimentarius parameters, which correspond to unsaturated fatty acids (91.67%). Moreover, this oil is characterized by the lowest acidity and peroxide indices, demonstrating its lipid stability against rancidity due to the lowest autoxidation products (ketones, aldehydes, hydroxyl alkenals, and dienes) formation which became off-flavor and toxic food for consumers associated with pro-oxidants action (processing manner, oxygen, heat, light, and metals) (9, 58). In addition, this oil presented a lower saponification index (141.80 mg KOH/g oil) compared with parameters and others obtained using petroleum solvent (188 and 189 mg KOH/g oil) (59), represented by the long-chain fatty acids (palmitic, stearic, oleic and linoleic acids), which can be used to identify these oils.

**Table 3:** Physicochemical characteristics of sunflower oil compared with Codex Alimentarius parameters.

**Parameters**	**Sunflower oil**	**Maximum values**
Peroxide index (mEq/kg)	16.61 ± 0.2	≤ 20 (55) (a)
Iodine index (g I_2_/100 g)	131.53 ± 0.1	118–141 (56) (b)
Acidity index (mg KOH/g)	0.54 ± 0.0	4.0 (56) (a)
Saponification index (mg KOH/g)	141.80 ± 1.92	184–196 (56) (b)
Relative density (20°C)	0.92 ± 0.1	0.92–0.93 (56) (c)

(a) Parameter for cold-pressed and virgin oils; (b) parameters for refined sunflower oils; (c) parameter for crude sunflower oil.

Relative density is the relevant parameter correlated with edible vegetable oil absorption and mass transfer rates during cooling or melting, better to lower values than the parameters (60). Sunflower oil obtained using a domestic machine presented a lower average (0.91) compared with that extracted using solvents (0.92–0.96) (61, 62). Although, these values represent a small difference among them, however, other parameters above referred can be used to identify the authenticity and origin of these oils.

### Determination of oxidative stability

**Figure 1:**
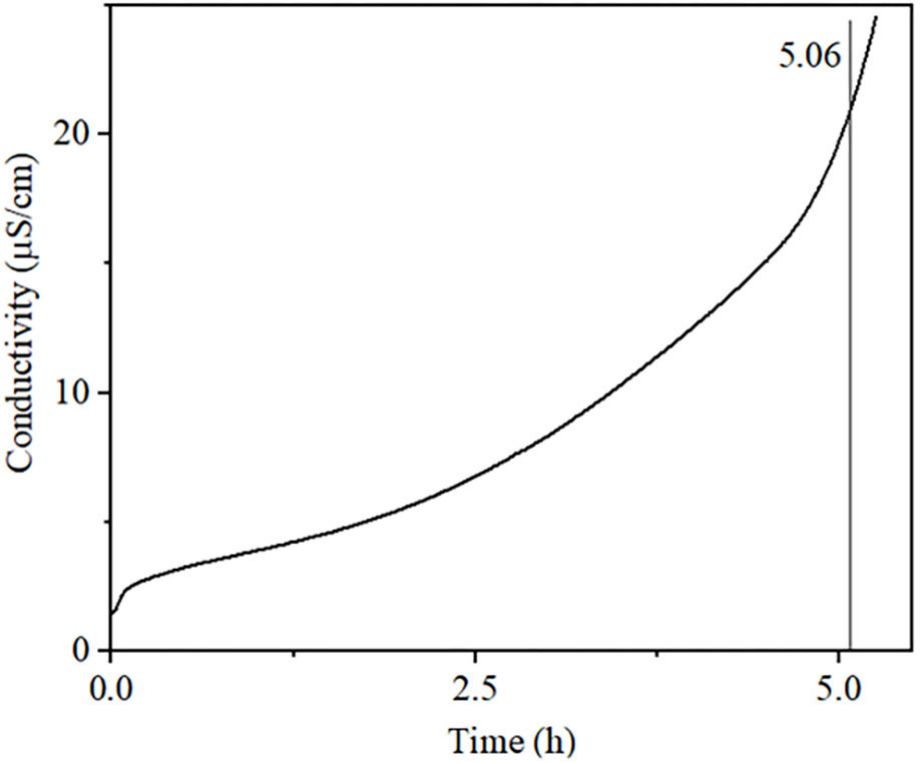
Conductivity vs. time determined by the Rancimat method. Oxidation stability of sunflower oil conducted at 110°C with an airflow of 10 L h^−1^.

Rancimat data revealed that the crude sunflower oil (unsaturation 91.67%) presented an induction period (IP) of 5.06 h (Figure 1). This behavior can be attributed to natural antioxidants in this oil as well observed to refined sunflower oil (unsaturation 88.40%), which the IP shifted from 5.5 to 7.5 h, respectively, in control and sunflower oil added polyphenol, subjected under a temperature of 110°C with airflow 20 L h^−1^ (63). Furthermore, the unsaturation amount of fatty acids is another relevant characteristic that is inversely proportional to oxidative stability. Sunflower hybrid oils (H) which presented higher iodine value (IV) showed lower IP, for instance, e.g., H19: (IV = 127, IP = 3.32 h) has lower IP compared with H21 (IV = 81, IP = 9.55 h) (64). Moreover, among unsaturation fatty acids, cold-pressed sunflower oil (SO) that presented a higher amount of oleic than linoleic fatty acids was more oxidative stably H20 (55), as well as SO1 (oleic, 86.52% and linoleic, 5.49%, IP = 19.87 h), while SO2 (oleic, 18.52% and linoleic, 66.02%, IP = 6.42 h) (65).

Therefore, to increase the stability of sunflower oil (rich in linoleic acid), it is recommended to blend it with oil rich in stable antioxidants such as tocopherols and thymoquinone or apply their natural antioxidants (17, 18).

### Thermogravimetric analysis/derivative thermogravimetriy, and differential scanning calorimetry

**Figure 2:**
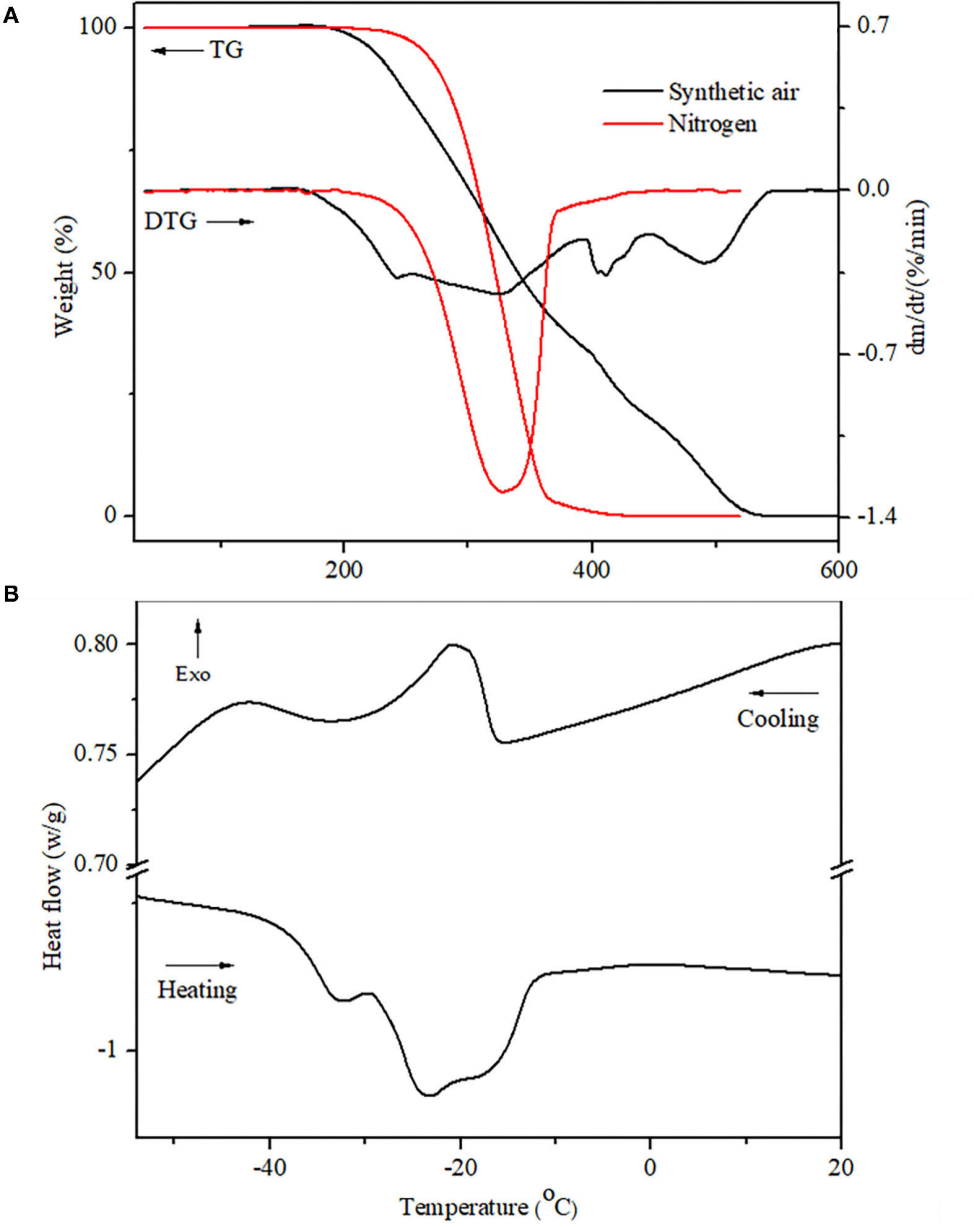
Thermal analyses of the sunflower oil. **(A)** TGA/DTG curves at 2°C min^−1^ heating from 10 to 550°C, under synthetic air and nitrogen atmospheres flow at 60 ml min^−1^ in dynamic conditions; **(B)** DSC curves of cooling and heating under the nitrogen atmosphere.

Thermogravimetric analysis/derivative thermogravimetry curves of crude sunflower oil in the presence of synthetic air and nitrogen atmospheres are shown in Figure 2. The TGA/DTG curves of sunflower oil submitted under a synthetic air atmosphere show three steps of thermal decomposition that are illustrated in Figure 2A and summarized in Table 4. The first step can be attributed to moisture linked by natural antioxidants (polyphenols, carotenoids, stanols, and vitamins A and C), dimers, trimmers, polymers, PUFAs, and other compounds formed from PUFAs (54.19%), represented by linoleic acid (54.00%). In addition, this process can be influenced by Zn (6,6228 mg/kg) and Fe (1.6637 mg/kg), both metals that negatively influence on oxidation stability (22, 26). The first stage of thermal stability is the most important, as it demonstrates that this cold-pressed sunflower oil can be heated up to 125°C without undergoing oxidative degradation.

**Table 4:** TGA/DTG curves of sunflower oil obtained under synthetic air and nitrogen atmospheres.

**Sample**	**Atmosphere**	**Steps**	**Temperature range (** **°** **C)**		**Event mass loss (%)**	**Residual mass (%)**
			**Initial**	**Final**		
Sunflower oil	Synthetic air	1^st^	126.16	254.34	15.48	0.21
		2^nd^	254.34	392.17	49.74	
		3^rd^	254.34	447.18	14.57	
	Nitrogen	1^st^	236.19	465.90	99.09	0.33

The second step of mass loss is attributed to products formed in the first step plus MUFAs (37.48%) decomposition represented by oleic fatty acid (37.29%), whose double bonds of MUFAs are broken and become SFAs of the oil. The third step of mass decomposition is attributed to the above fatty acids plus SFAs (7.26%) (palmitic (4.13%), stearic acids (3.13%), and waxes (C36–C48), of which the majority are represented by C36, C37, C40, and C41, as previously reported in cold-pressed sunflower oil (49). The minor last mass loss is attributed to carbonaceous residue substances. This behavior was reported in commercial sunflower oil, although with more thermal stability compared with the current study (66–68). Moreover, cold press and unrefined oil present more oleic than refined one (10), natural antioxidants (69) and minerals, which are healthy and regulators of several metabolic diseases (9, 68, 70), and economic and ecological benefits from food by-products (71) than refined ones. Moreover, synthetic antioxidants, butylated hydroxyanisole (BHA), butylated hydroxytoluene (BHT), tert-butylhydroquinone (TBHQ), and others (10, 72) replace natural antioxidants in industrial edible vegetable oils, which are associated with the prevalence of metabolic diseases (cellular damage, cancers) and environmental contamination (73, 74).

In the nitrogen atmosphere, the oil showed one high step mass decomposition justified by a higher amount of unsaturated fatty acids (oleic and linoleic fatty acids) compared with SFAs. The minor last mass loss is attributed to formed carbonaceous residual substances.

In Figure 2B, DSC curves show two exothermic crystallization peaks, which the first attributed to SFAs and MUFAs, with T_onset_ at −16.57°C and enthalpy peak at 1.979 J/g, followed by a second peak representing PUFAs observed at −34.52°C, with enthalpy peak at 1.466 J/g. Moreover, heating the sunflower oil, two peaks were also observed, the first peak corresponding to MUFAs, whose T_onset_ was observed at −36.27°C and enthalpy at 1.866 J/g. The T_onset_ of the second peak appeared at −28.07°C and the enthalpy peak at 24.59 J/g.

In the current study, DSC results were observed in lower temperatures than commercial sunflower oil due to their fatty acids and natural antioxidant composition, which influence its thermal oxidative levels (75). The TGA and DSC analyses are used to qualify, authenticate, and recognize vegetable oils, thus avoiding their adulteration, falsification, and consumption of improper products.

### Optical molecular analyses: UV-Visible absorption and fluorescence spectroscopy

**Figure 3:**
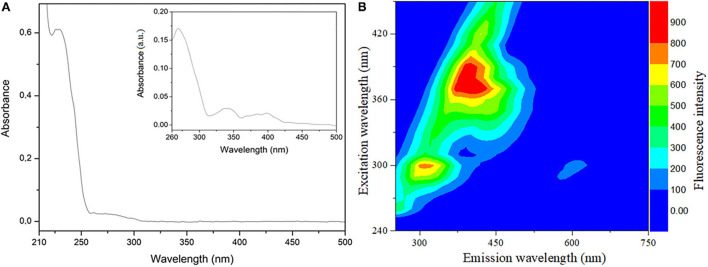
Optical molecular analysis used sunflower oil diluted in hexane HPLC 99.9% at 1 × 10^−3^ g L^−1^. **(A)** UV-Visible absorption spectrum wavelength collected between 200 and 800 nm and the inset “zoom”; **(B)** Emission-excitation fluorescence spectrum map with excitation obtained between 240 and 450 nm and emission (250–750 nm).

Figure 3A illustrates sunflower oil UV-Vis absorption spectrum with two major absorbance regions one in the 223–236 nm range and the other from 257 to 452 nm (inset of Figure 3A). The first absorption band can be attributed to phytocholesterols (phytostanols), phytosterols, and tocopherols (vitamin E). The second region corresponds to carotenoids and fatty acids (linoleic, oleic acids) (6, 76). The consumption of oils rich in phytocholesterols, carotenoids, tocopherols and phytosterols, polyphenols, and unsaturated fatty acids are widely correlated with avoiding and regulating several metabolic diseases (3, 9).

The UV-Vis is used to monitor oxidation, identity, and authenticity, as the absorption in the 400–520 nm range appears higher in adulterated sunflower oil (77).

Figure 3B shows the excitation–emission map of sunflower oil exhibiting two intense bands. The first region presents emissions in the 297–327 nm range when excited between 290 and 310 nm. The second region of emission is observed between 360 and 445 nm due to excitation in the 360–400 nm range. These fluorescence bands can be correlated with the presence of vitamin E (tocopherols and tocotrienols), carotenoids, chlorophyll, and unsaturated fatty acids in sunflower oil (78).

The fluorescence emission wavelength ranging from 400 to 500 is used to identify, qualify, and authenticate original and adulterated vegetable edible oils due to the oxidation of fatty acids products and tocopherols (79).

### Trace elements concentration in sunflower oil

Table 5 summarizes the data of six trace elements quantified in sunflower oil compared with Codex Alimentarius contents for oils and dietary reference intakes (DRIs).

**Table 5:** Trace elements in sunflower oil quantified by ICP OES (mg/kg ± SD) compared with nutritional recommendations for adult, pregnancy, lactation, and children by RDA/AI and FAO/WHO.

**Trace elements**	**Concentration (mg/kg)**	**FAO/WHO (mg/kg)**	Dietary reference intakes (DRI) and adequate intake (AI^*^) (mg/day) **(****80****)**								
			**Children**	**Males**		**Females**		**Pregnancy**		**Lactation**	
			**8 y. old**	**18 y. old**	**30 y. old**	**18 y. old**	**30 y. old**	**18 y. old**	**30 y. old**	**18 y. old**	**30 y. old**
Cr	0.0242 ± 0.0049	0.002 (81)	0.015^*^	0.035^*^	0.035^*^	0.024^*^	0.025^*^	0.029^*^	0.030^*^	0.044^*^	0.045^*^
Mn	0.2372 ± 0.0051	3.00 (82)	1.5^*^	2.2^*^	2.3^*^	1.6^*^	1.8	2.0^*^	2.0^*^	2.6^*^	2.6^*^
Fe	1.6637 ± 0.0393	14.00 (82)	10	11	8	15	18	27	27	10	9
Cu	0.2791 ± 0.0094	0.90 (82)	0.44	0.89	0.9	0.89	0.9	1	1	1.3	1.3
Zn	6.6228 ± 0.0788	15.00 (82)	5	11	11	9	8	12	11	13	12
Al	1.0401 ± 0.0199	5.00 (83)	ND	ND	ND	ND	ND	ND	ND	ND	ND

Elements Mg, Co, Ni, Cd, Pb, As, and Se < LOD; NE, not established; ND, not determined;

* The value for AI is used when there are no calculated values for the RDA.

Beyond the endogenous biological processes, plants acquire minerals from the soil, besides exogenous processes (environment pollution by industries, transports, mechanization, fertilizers, and pesticides used in agriculture), which influence raw material for edible plant production (42). Moreover, minerals can contaminate edible vegetable oils during production, refining, and storing processes.

Minerals (Ag, As, Be, Ca, Cu, Zn, Fe, Mg, Mn, Cd, Co, Na, K, Ni, Pb, and V) are used to monitor and qualify edible vegetable oils: corn (*Zea mays*), hazelnut (*Corylus avellana*), olive (*Olea europaea*), and sunflower in Turkey (84), pequi (*Caryocar brasiliense*), primrose (*Oenothera biennis*), avocado (*Persea americana*), coconut (*Cocos nucifera*), grape seed (*Vitis vinifera*), babassu (*Attalea speciosa*), and licuri (*Syagrus coronata*) in Brazil (40), and several varieties in China (26) and Iran (24).

Mineral concentrations can be used as a relevant fingerprint to distinguish the provenance, originality, and adulterated edible vegetable oils, promoting health benefits. The average concentrations of trace elements quantified in sunflower oil in decreased order are of Zn (6.6228 mg/kg) > Fe (1.6637 mg/kg) > Al (1.0401 mg/kg) > Cu (0.2791 mg/kg) > Mn (0.2372 mg/kg) > Cr (0.0242 mg/kg). Only Cr concentrations exceeded 1,210% of FAO/WHO limits, which can be influenced by the vehicle fumes, fertilizers, and pesticides used, as reported in the intensive agriculture modern farming, as well as the cultivated areas located near the road with high vehicle traffic (42, 85). In contrast, Zn, Fe, Al, Cu, and Mn concentrations were lower than FAO/WHO and DRI/AI^*^ limits. Moreover, Cr and Zn concentrations were within DRI/AI^*^ limits, while Cu, Fe, and Mn concentrations were quantified lower than DRI/AI^*^ limits (Table 5).

Furthermore, Al concentration was higher than quantified in industrial refined oils of avocado, primrose, babassu, licuri, pequi, olive, and grape seeds (0.04–0.52 mg kg^−1^) (32). In contrast, Zn, Cu, and Mn amounts were reported between those found in commercially refined oils of sunflower, olive, canola, soybean, corn, and hazelnut corresponding to 1.03–9.54, 0.05–4.504, and 0.04–1.76 mg kg^−1^, respectively. The concentrations of Cr and Fe were lower than reported in sunflower, olive, canola, soybean, corn, and hazelnut oils corresponding to 0.0126–7.106 and 7.78–28.93 mg kg^−1^, respectively (24, 84, 86).

Thus, in light of the acceptable concentrations of trace elements regarding referential parameters, the consumption of the sunflower oil herein studied can be beneficial, because Cr, Cu, Fe, Mn, and Zn play essential physiological roles in defense response, protein construction, enzymatic reactions, signaling pathways, regulation of oxidative stress and metabolic diseases, and others (87). However, lower Al concentrations are associated with reduced occurrence of metabolic diseases and dysfunctions, such as cancers, Alzheimer's and Parkinson's diseases, inhibited enzymatic, cytotoxic and neurotoxic reactions, gut imbalance, skeletal disorders, and others (42).

### Health risk assessment

Most studies reported daily consumption of vegetable oils from 25 to 30 g kg^−1^ (26, 88). Other studies demonstrated that quantities of cooking oils depend on the type of dishes: pure vegetables range from 9 to 167 g kg^−1^, with a mean of 56 g kg^−1^, pure meat from 4 to 353 g kg^−1^ (142 g kg^−1^), and mixed meat-vegetable 7 to 394 g kg^−1^ (110 g kg^−1^) (89).

However, the calculated quantities of the sunflower oil daily intake (g kg^−1^) in comparison with other studies based on the concentration of trace elements regarding acceptable non-carcinogenic risk (CR) < 10^−6^ to 10^−4^ and total non-carcinogenic hazard index (HI) < 1 are summarized in Table 6. Based on the country of origin, it is remarkable that the quantity of sunflower oil daily intake is independent of the obtained by cold-pressed (Brazil and Romania) or petroleum solvent extraction (China and Turkey). Regarding trace elements concentrations in sunflower oil, the quantity of sunflower oil daily intake by individuals aged 8, 18, and 30 years old are, respectively, described in decreased order for Brazil (0.61, 1.46, 1.65 g/day) > China (0.41, 0.99, 1.12 g/day) > Romania (0.037, 0.08, 0.099 g/day) > Turkey (0.0097, 0.0234, 0.0264 g/kg) > Turkey (0.0093, 0.0224, 0.0253 g/kg).

**Table 6:** Non-carcinogenic risk (CR), hazard quotient (HQ), and total non-carcinogenic hazard index (HI) of trace elements on ingestion rate (IR g/kg) of sunflower oil obtained by cold-press (Brazil and Romania) and commercially available (China and Turkey).

**Study**	**Years old**	**IR (g/day)**	**Index**	**Trace elements**														**HI**
				**Co**	**Cr**	**Cu**	**Fe**	**Mn**	**Ni**	**Pb**	**Se**	**Zn**	**Li**	**Mo**	**Cd**	**As**	**Al**	
Brazil (Current study)	8	0.61	CR	-	2.8 × 10^−4^	-	-	-	-	-	-	-	-	-	-	-	-	
			HQ	0	0.18926	0.1637	0.05576	0.03975	0	0	0	0.51794	-	-	0	0	0.0244	0.99
	18	1.46	CR	-	2.9 × 10^−4^	-	-	-	-	-	-	-	-	-	-	-	-	
			HQ	0	0.18996	0.16431	0.05597	0.0399	0	0	0	0.51985	-	-	0	0	0.02449	0.99
	30	1.65	CR	-	2.9 × 10^−4^	-	-	-	-	-	-	-	-	-	-	-	-	
			HQ	0	0.19014	0.16447	0.05602	0.03994	0	0	0	0.52036	-	-	0	0	0.02452	0.99
Romania (32)	8	0.037	CR	-	3.6 × 10^−4^	-	-	-	-	-	-	-	-	-	-	-	-	
			HQ	-	0.2372	0.007	0.00002	0.00793	0.01352	-	0.333	0.00043	0.14231	0.25615	-	-	-	0.99
	18	0.08	CR	-	3.2 × 10^−4^	-	-	-	-	-	-	-	-	-	-	-	-	
			HQ	-	0.21505	0.00677	0.00002	0.00719	0.01226	-	0.30194	0.00039	0.12903	0.23226	-	-	-	0.99
	30	0.099	CR	-	3.5 × 10^−4^	-	-	-	-	-	-	-	-	-	-	-	-	
			HQ	-	0.23571	0.00743	0.00002	0.00788	0.01344	-	0.33094	0.00042	0.14143	0.25457	-	-	-	0.99
China (26)	8	0.419	CR	-	-	-	-	-	-	1.4 × 10^−6^	-	-	-	-	2.1 × 10^−5^	2.7 × 10^−5^	-	-
			HQ	-	-	0.02659	0.67224	0.05122	0.0274	0.04029	-	0.06607	-	-	0.056565	0.05909		0.99
	18	0.99	CR	-	-	-	-	-	-	1.4 × 10^−6^	-	-	-	-	2.1 × 10^−5^	2.6 × 10^−5^	-	
			HQ	-	-	0.02635	0.66608	0.05075	0.02715	0.03992	-	0.06547	-	-	0.05605	0.05855	-	0.99
	30	1.128	CR	-	-					1.4 × 10^−6^	-	-	-	-	2.1 × 10^−5^	2.7 × 10^−5^	-	
			HQ	-	-	0.02659	0.67220	0.05122	0.02739	0.04029	-		-	-	0.05656	0.05909	-	0.99
Turkey (84)	8	0.0093	CR	-	-	-	-	-	-	3 × 10^−5^	-	-	-	-	5.1 × 10^−7^	-	-	
			HQ	0.0065	-	0.00099	0.05435	0.03097	-	0.90323	-	0.00132	-	-	0.00134	-	-	0.99
	18	0.0224	CR	-	-	-	-	-	-	3.1 × 10^−5^	-	-	-	-	5.2 × 10^−7^	-	-	
			HQ	0.10074	-	0.01539	0.8419	0.0048	-	0.01399	-	0.00133	-	-	0.000000021	-	-	0.99
	30	0.0253	CR	-	-	-	-	-	-	3.1 × 10^−5^	-	-	-	-	5.2 × 10^−7^	-	-	
			HQ	0.00651	-	0.0099	0.05437	0.03098	-	0.90357	-	0.02043	-	-	0.001358971	-	-	0.99
Turkey (86)	8	0.0097	CR	-	1.3 × 10^−3^	-	-	-	-	3.2 × 10^−7^	-	-	-	-	8.1 × 10^−6^	-	-	
			HQ	-	0.88369	0.02766	0.00475	-	0.03231	0.00933	-	0.0079	-	-	0.02127	-	-	0.99
	18	0.0234	CR	-	1.3 × 10^−3^	-	-	-	-	3.2 × 10^−7^	-	-	-	-	8.2 × 10^−6^	-	-	
			HQ	-	0.89398	0.02799	0.00481	-	0.03268	0.00944	-	0.00799	-	-	0.02151	-	-	0.99
	30	0.0264	CR	-	1.3 × 10^−3^	-	-	-	-	3.2 × 10^−7^	-	-	-	-	8.2 × 10^−6^	-	-	
			HQ	-	0.89333	0.02797	0.00481	-	0.03266	0.00943	-	0.00799	-	-	0.0215	-	-	0.99

Thus, given the findings of the current study (mineral concentrations), it seems relevant to explore the calculating amount of vegetable oil consumption per day based on trace elements quantified from different origins to be used for health promotion and regulation of several metabolic diseases.

## Conclusion

The assessed sunflower oil obtained by a domestic cold-pressing demonstrates optimal qualitative properties for consumption, correlated with observed results on fatty acids composition, physicochemical optical features, thermal and oxidative qualities, and trace elements compared with DRI/AI^*^ and FAO/WHO parameters herein evaluated. However, Cr concentration in sunflower oil was above FAO/WHO limits, which can be used as an indicator of ambient pollution.

However, to obtain values of HI < 1, and CR < 10^−4^, the maximum sunflower oil daily consumption varies between 0.61, 1.46, and 1.65 g kg^−1^, respectively, for individuals aged 8, 18, and 18 years. Moreover, the calculated results based on trace elements concentration regarding HI < 1 and CR < 10^−4^ indices of the sunflower oil previously qualitative approved show lower daily intake compared with prior daily consumption varying from 25 to 142 g kg^−1^.

Thus, it is expected that this quantitative daily consumption of sunflower oil here presented can be used for other vegetable oils and several foodstuffs for health improvement and metabolic disease regulation.

## Data availability statement

The raw data supporting the conclusions of this article will be made available by the authors, without undue reservation.

## Author contributions

DM: conceptualization of the topic, methodology, investigation, formal analysis, writing the original draft, visualization, and data curation. EM, FM, LO, and AC: methodology. DB, AP, LC, KF, PH, MV, RO, and RG: investigation and formal analysis. VN and DM: writing, reviewing, and editing. VN: supervision, funding acquisition, and project administration. All authors have read and approved the final version of the manuscript, ensure the accuracy and integrity of the work, and agree to be accountable for all appearances.

## Funding

This research was funded by the Federal University of Mato Grosso do Sul (UFMS), the Coordination of Higher Education Personnel Improvement (CAPES)-Portaria 2016/2018 (CAPES-finance code 001), and the Brazilian Research Council (CNPq) (CNPq: Process No 310621/2020-8). The Ph.D scholarship was financially supported by the Programa MAI-DAI/UFMS: inovações para o agronegócio e a sustentabilidade ambiental: Acordo: 25/2022 Universidade Federal de Mato Grosso do Sul–Empresa Brasileira de Pesquisa Agropecuária (UFMS-EMBRAPA) and the National Council for Scientific and Technological Development (Conselho Nacional de Desenvolvimento Científico e Tecnológico (CNPq) processo: 403651/2020-4.

## Conflict of interest

The authors declare that the research was conducted in the absence of any commercial or financial relationships that could be construed as a potential conflict of interest.

## Publisher's note

All claims expressed in this article are solely those of the authors and do not necessarily represent those of their affiliated organizations, or those of the publisher, the editors and the reviewers. Any product that may be evaluated in this article, or claim that may be made by its manufacturer, is not guaranteed or endorsed by the publisher.
